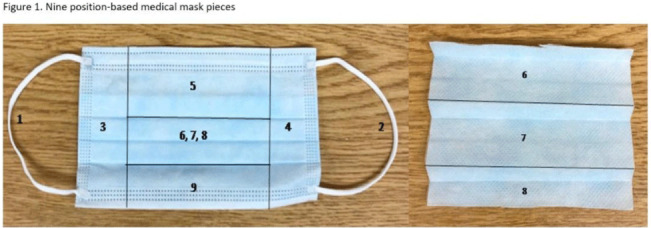# SARS-CoV-2 N95 contamination worn under a face shield, via medical mask surrogate, in healthcare providers treating COVID-19

**DOI:** 10.1017/ash.2022.127

**Published:** 2022-05-16

**Authors:** Amanda Graves, Bobby Warren, Aaron Barrett, Sarah Lewis, Becky Smith, David Weber, Emily Sickbert-Bennett Vavalle, Deverick Anderson

## Abstract

**Background:** SARS-CoV-2 N95 mask contamination in healthcare providers (HCPs) treating patients with COVID-19 is poorly understood. **Method:** We performed a prospective observational study of HCP N95 respirator SARS-CoV-2 contamination during aerosol-generating procedures (AGPs) on SARS-CoV-2–positive patients housed in a COVID-19–specific unit at an academic medical center. Medical masks were used as surrogates for N95 respirators to avoid waste and were worn on top of HCP N95 respirators during study AGPs. Study masks were provided to HCPs while donning PPE and were retrieved during doffing. Additionally, during doffing, face shields were swabbed with Floq swabs premoistened with viral transport media (VTM) prior to disinfection. Medical masks were cut into 9 position-based pieces, placed in VTM, vortexed, and centrifuged (Fig. 1). RNA extraction and RT-PCR were completed on all samples. RT-PCR–positive samples underwent cell culture infection to detect cytopathic effects (CPE). Contamination was characterized by mask location and front and back of face shields. Patient COVID-19 symptoms were collected from routine clinical documentation. Study HCPs completed HCP-role–specific routine care (eg, assessing, administering medications, and maintaining oxygen supplementation) while in patient rooms and were observed by study team members. **Results:** We enrolled 31 HCPs between September and December 2021. HCP and patient characteristics are presented in Table 1. In total, 330 individual samples were obtained from 31 masks and 26 face shields among 12 patient rooms. Of the 330 samples, 0 samples were positive for SARS-CoV-2 via RT-PCR. Positive controls were successfully performed in the laboratory setting to confirm that the virus was recoverable using these methods. Notably, all samples were collected from HCPs caring for COVID-19 patients on high-flow, high-humidity Optiflow (AGP), with an average of 960 seconds (IQR, 525–1,680) spent in each room. In addition to Optiflow and routine care, study speech pathologists completed an additional AGP of fiberoptic endoscopic evaluation of swallowing. Notably, 29 (94%) of 31 study HCP had physical contact with their patient. **Conclusions:** Overall, mask contamination in HCPs treating patients with COVID-19 undergoing AGPs was not detectable while wearing face shields, despite patient contact and performing AGP.

**Funding:** None

**Disclosures:** None

**Table:**
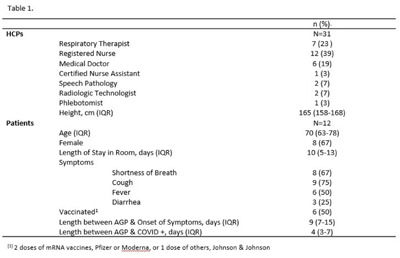

**Figure:**